# Report on Leg Sensilla of Notonectidae (Hemiptera, Heteroptera)

**DOI:** 10.3390/insects16101048

**Published:** 2025-10-14

**Authors:** Meng-Yao Fan, Tong-Yin Xie

**Affiliations:** College of Plant Protection, Northeast Agricultural University, Harbin 150030, China; neau_fanmy@163.com

**Keywords:** leg sensilla, Notonectidae, Hemiptera, Heteroptera

## Abstract

**Simple Summary:**

The purpose of this study was to analyze the morphological types and arrangement of leg sensilla in *Anisops*, *Enithares* and *Notonecta*, which belong to Notonectidae. In this study, we examined seventeen species from three genera of Notonectidae and found ten types of sensilla on the legs. Sensilla arch-shaped (SAr) and sensilla spoon-shaped (SSp) were reported for the first time.

**Abstract:**

Notonectidae belongs to the infraorder Nepomorpha within the order Hemiptera. The aim of this study was to analyze the morphological types and arrangement of leg sensilla in *Anisops*, *Enithares* and *Notonecta*. A variety of sensilla are distributed on the legs. These sensilla are responsible for receiving signals from the external environment. Mechanoreceptors exhibit the highest diversity. Using a scanning electron microscope, ten types of sensilla were identified on the legs of seventeen species from Notonectidae. Basic types of mechanoreceptors, including sensilla trichodea (ST1, ST2), sensilla chaetica (SCh1, SCh2), sensilla basiconica (SB2) and sensilla campaniformia (SCa), were distributed across all the studied species. In *Anisops*, sensilla arch-shaped (SAr) and sensilla spoon-shaped (SSp) were reported for the first time. Additionally, six subtypes of ST were distinguished in *Anisops*, among which ST3, ST4, ST5 and ST6 are unique. In *Enithares* and *Notonecta*, sensilla styloconica (SS) were observed; these sensilla are hypothesized to function as both mechanoreceptors and gustatory receptors. Beyond mechanoreceptors, we also identified thermo-hygroreceptors—sensilla ampullacea (SA) and sensilla coeloconica (SCo)—as well as a potential olfactory sensilla type, namely, sensilla placodea multilobated (SPM). These findings suggest that Notonectidae leg sensilla play an important role in the perception of aquatic environments and prey localization.

## 1. Introduction

Aquatic insects serve as a vital food source for both aquatic vertebrate and invertebrate predators; some of them act as natural predators and prey on mosquitoes [1,2]. They play a crucial role in aquatic food webs [3]. Aquatic Hemiptera can serve as bioindicators for water pollution and biocontrol agents in aquatic ecosystems [4]. The species of Notonectidae are commonly known as backswimmers for swimming with their backs facing down, using their long posterior legs as oars to propel themselves through the water [5]. Notonectidae is a cosmopolitan family and ranks second in diversity among aquatic bugs, with approximately 400 species and 11 genera in the world [6,7]. In China, 4 genera and 32 species have been recorded [8].

Insects mainly find hosts and conspecifics using chemosensory signals [9]. Sensilla systems are especially important for aquatic insects that live in dense, dark, and highly complex habitat conditions or species with poor vision capabilities, as they can perceive different types of stimuli through these sensilla [10,11,12]. Chemoreception in aquatic insects is the perception of chemicals from organic or inorganic sources: those in aqueous solutions are sensed by gustatory sensilla, while airborne ones are detected by olfactory sensilla. Like other aquatic animals, aquatic insects have a vague distinction between taste and olfaction, but this differentiation is still used based on the sensillum structure, response, location or the insect’s behavioral response [13,14]. These sensilla are composed of cuticular parts, sheath cells and sensory neurons [14]. Mechanoreceptive, chemoreceptive and thermo-hygroreceptive sensilla are distinguished based on their internal and external structures. Sensilla that possess pores are usually chemoreceptive sensilla, while sensilla with a surface without pores are mechanoreceptive or thermo-hygroreceptive sensilla [15,16]. Sensilla play an important role in a number of behaviors, such as host recognition, mate location, oviposition, aggregation and defense [17].

The morphology and function of the sensilla on insect legs are closely related to their survival strategies. In recent years, researchers have made significant progress in aspects such as their types, functions and mechanisms. Legs play important roles in the chemoreception of insects [18]. The legs of *Drosophila* are involved in making preliminary contact with food resources and non-volatile pheromones [19,20]. The tarsal gustatory sensilla of *Helicoverpa armigera* play a crucial role in perceiving sugar substances present in floral nectar, and the chemosensory information they transmit determines the feeding behavior of the insect; meanwhile, the response of tarsal taste receptor neurons to lysine may be associated with the oviposition behavior of *Helicoverpa armigera* [21]. Some studies suggest that the sensilla on the legs may play a dual role in taste or olfaction [18]. Research on taste recognition in insects via the gustatory sensilla on the tarsi has been relatively in-depth [22].

In aquatic Heteroptera, the labial and the antennal sensilla of an array of taxa were already extensively studied, providing a good basis for comparative research [15,23,24,25,26,27,28]. Sensilla exhibit extraordinary diversity in form and function. Six types of sensilla have been observed on the antennae of Notonectidae, namely, sensilla trichodea (ST), sensilla chaetica (SCh), sensilla campaniformia (SCa), sensilla basiconica (SB), sensilla coeloconica (SCo) and sensilla ampullacea (SA).

Backswimmers detect prey through both visual and tactile mechanisms [29]. The surface of insects’ legs is equipped with various sensory structures, primarily serving mechanoreceptive and chemoreceptive functions [30,31]. Mechanoreceptors provide accurate information on prey location by detecting water waves, enabling backswimmers to distinguish between prey and non-prey organisms [32]. These structures remain to be studied in the leg sensilla of the aquatic bugs. Existing studies have insufficient coverage of families and genera.

Up to now, morphological studies on leg sensilla have only been conducted on 12 species from Corixidae (4 species), Ochteridae (2 species) and Gelastocoridae (6 species), within the infraorder Nepomorpha, to analyze their associations with habitat environments [16]. The studies identified eight main types of sensilla, namely, sensilla trichodea (ST), sensilla chaetica (SCh), sensilla campaniformia (SCa), sensilla basiconica (SB), sensilla placodea multilobated (SPM), sensilla coeloconica (SCo), sensilla ampullacea (SA) and sensilla styloconica (SS). The results indicate that the morphology and distribution of sensilla are closely related to habitats. However, all groups exhibit a high diversity of mechanoreceptive sensilla, which supports the hypothesis that there are specialized sensilla in Nepomorpha [16].

Species from Notonectidae are generalized predators, hunting on sight or vibrations on the water surface. Prey is grabbed and handled with the foreleg and midleg [33]. We studied sensilla on the legs of seventeen species belonging to *Anisops*, *Enithares* and *Notonecta* from Notonectidae. At the same time, we also speculated on the role of leg sensilla. Further ultrastructural studies are needed to validate functional differences.

## 2. Materials and Methods

### 2.1. Materials

The experimental insects were mainly collected from Guangdong, Guizhou, Heilongjiang, Inner Mongolia Autonomous Region and Yunnan in mainland China. This research focused on the leg sensilla morphology of seventeen species belonging to three genera (Table 1).

### 2.2. Scanning Electron Microscope (SEM) Characterization

Seventeen species of Notonectidae were dissected using a ZEISS SteREO Discovery. V20 stereomicroscope (ZEISS, Oberkochen, Germany), tweezers and dissecting needles. Their legs were removed and fixed in a 2.5% glutaraldehyde solution (Biosharp, Beijing, China) at 4 °C for 24 h in a dark, refrigerated environment. After fixation, the samples underwent ultrasonic cleaning for 20 min. They were then rinsed 3 times with a phosphate buffer solution (pH 7.2), each for 15 min, and dehydrated through an ethanol (Tianli, Tianjin, China) gradient series (75%, 80%, 90%, 95% and 3 changes of 100% ethanol), with each step lasting 15 min. The dehydrated samples were air-dried at room temperature for 12 h. For electron microscopy, the legs were mounted on specialized stages with conductive adhesive, using dissecting needles and forceps under microscopic guidance to ensure proper orientation. The mounted specimens were gold-coated with a sputtering coater and examined under a Hitachi TM4000 desktop scanning electron microscope (Tokyo, Japan) at an acceleration voltage of 15 kV and a magnification range of 20×–3000×. This examination took place at the large-scale instruments and equipment sharing service platform of Northeast Agricultural University in Harbin [34]. We follow the terminology and classification reported in other papers on the sensilla of insects [16,23,24].

## 3. Results

### 3.1. Gross Morphology of the Legs

**Figure 1:**
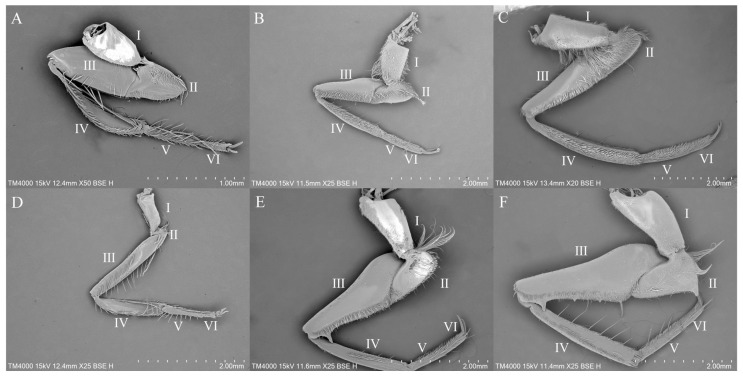
Morphology of the foreleg (**A**–**C**) and midleg (**D**–**F**): *Anisops exiguus* (**A**); *Enithares ciliata* (**B**); *Notonecta montandoni* (**C**); *Anisops bouvieri* (**D**); *Enithares biimpressa* (**E**); *Notonecta triguttata* (**F**). I—coxa; II—trochanter; III—femur; IV—tibia; V, VI—tarsus.

On the forelegs of the species from the three genera under study (Figure 1A–C), the coxae are short and stout with hairy surfaces. The species in this study exhibit a small cluster of thin hairs near the trochanters. On the femora of *Enithares* and *Notonecta*, there are more sensilla distributed on the ventral side, while the femoral surfaces of *Anisops* have fewer sensilla. The tibia of *Anisops* is flattened, whereas the tibiae of *Enithares* and *Notonecta* are slender and cylindrical. Both the tibiae and tarsi of *Enithares* and *Notonecta* are densely covered in neatly arranged hairs. In *Anisops*, males possess a single tarsal segment on the foreleg, whereas females have two segments. The tarsi of the foreleg of *Enithares* and *Notonecta* consist of two segments.

Regarding the midlegs of the three genera (Figure 1D–F), those of *Anisops* exhibit a relatively slender overall morphology, whereas the midlegs of *Enithares* and *Notonecta* are more robust. All three genera possess short hairs on their coxae. The coxae of *Anisops* are relatively slender, and sensilla occupy most of the surface of the coxae. A small tuft of thin hairs is present near the trochanters in all three genera. The femora of *Enithares* and *Notonecta* are enlarged and highly curved, with a protrusion near the proximal end. The tibiae of *Anisops* are flat, while the tibiae of *Enithares* and *Notonecta* are cylindrical. The tibiae and tarsi of *Enithares* and *Notonecta* are covered with densely arranged short, thick hairs, in contrast to the sparse hairs on the corresponding segments of *Anisops*. The midlegs of *Anisops* have sparse surface hairs, whereas those of *Enithares* and *Notonecta* are densely covered with hairs.

**Figure 2:**
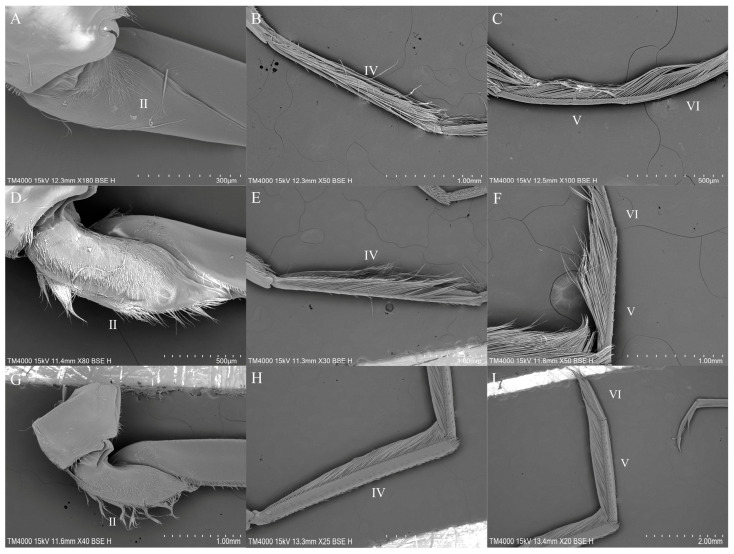
Morphology of the hindleg: *Anisops exiguus* (**A**,**B**); *Anisops bouvieri* (**C**); *Enithares tibialis* (**D**); *Enithares biimpressa* (**E**); *Enithares sinica* (**F**); *Notonecta triguttata* (**G**); *Notonecta chinensis* (**H**,**I**). II—trochanter; IV—tibia; V, VI—tarsus.

The hindlegs, as swimming appendages, are significantly longer than their forelegs and midlegs. While the hindlegs of *Anisops* are relatively slender, the hindlegs of *Enithares* and *Notonecta* are more robust. The trochanters of *Anisops* are small and approximately conical (Figure 2A), with the distal half tapering sharply, while the trochanters of *Enithares* and *Notonecta* are larger (Figure 2D,G). The femora and tibiae of the three genera are club-shaped. Swimming hairs are present on the ventral side of both the tibiae and tarsi (Figure 2B,C,E,F,H,I). The tarsus of *Anisops* is gradually curved (Figure 2C). The tarsi of *Enithares* and *Notonecta* are straight (Figure 2F,I).

### 3.2. Morphology and Types of Leg Sensilla

Ten main types of sensilla were identified on the legs of the studied species (Table 2).

1. Sensilla trichodea (ST)—Long, thin, hairlike sensilla, with smooth or ribbed surfaces. They have a flexible socket (a thin membrane connects the cuticle of the leg with the cuticle of the sensillum, making it movable at the base), which gives them a putative mechanoreceptive function. The shape of this sensillum varies from tapered at the top to flattened at the top. The tip is either straight or bent. This type of sensilla appears in groups, covering large areas of the surface. The other function performed by sensilla trichodea is gustation. In this case, the sensillum occurs with a single pore on the tip.
  a. ST1—Long, thin, hairlike sensilla with a ribbed surface, without pores—they perform a mechanoreceptive function.
  b. ST2—Long, thin, hairlike sensilla with a smooth surface, without pores—they perform a mechanoreceptive function.
  c. ST3—Long, flattened sensilla. Their bases are cylindrical and gradually flatten toward the tips. Their surfaces have more or less distinct stripes with no pores present—they perform a mechanoreceptive function.
  d. ST4—Long, flattened, sometimes curved inwards sensilla. The surface has shallow stripes, without pores—they perform a mechanoreceptive function.
  e. ST5—Long ribbed sensilla, flattening and widening along the length, with a ribbed frayed end resembling a brush, without pores—they perform a mechanoreceptive function.
  f. ST6—Their bases are flattened and gradually widen toward the obtusely rounded tips. These types of sensilla also perform a mechanoreceptive function.
2. Sensilla chaetica (SCh)—Thick sensilla with pronounced ribs on the surface. The length varies. The tip is either pointed or rounded. It has a flexible socket, like sensilla trichodea, but it is easily distinguished from this other type because it is visibly thicker and more rigid. This type is also described in the literature as mechanoreceptive sensilla.
  a. SCh1—These sensilla are thick, rigid, and marked with distinct stripes. They taper from a thicker base to a sharply pointed or slightly rounded tip.
  b. SCh2—These sensilla are thick and rigid, with pronounced ribs on the surface. The sensilla are short and slightly bent.
3. Sensilla campaniformia (SCa)—Oval or elongated disks lying flat on the surface, usually with a visible pore in the middle. This type also has a flexible socket. These sensilla belong to mechanoreceptors and are described as pressure sensilla.
4. Sensilla basiconica (SB)—Conical structure, usually smaller than the sensilla trichodea, with porous or non-porous surfaces and a flexible or inflexible socket.
  a. SB1—These sensilla are relatively large, and the socket is flexible. The surface is smooth, without any streaks, and the tips are obtusely rounded. They are usually distributed on the tibia.
  b. SB2—The sockets of these sensilla are flexible. The sensilla are relatively small and taper gradually from their bases to the tips. Their surfaces have striations. They are the smallest type among the sensilla basiconica.
  c. SB3—These sensilla have stripes on their surfaces and sharp ends, are short in size, and usually cling closely to the surface of the leg.
  d. SB4—These are conical structures that are either straight or branched at the base, gradually converging towards the tip. They have a smooth surface and a flexible socket.
5. Sensilla coeloconica (SCo)—Small cone-like with an inflexible socket and smooth surfaces. Individually distributed or concentrated in smooth areas of the legs, they are considered to have a thermo-hygroreceptive function.
6. Sensilla ampullacea (SA)—These sensilla have thermo-hygroreceptive functions. Their sockets are inflexible and hidden in a deep cavity. Only a circular opening can be seen on the surface.
7. Sensilla styloconica (SS)—Pegs arise from a bulge of cuticle, each peg with a flexible socket and a ribbed surface. These sensilla are believed to perform mechanoreceptive or gustatory functions.
8. Sensilla placodea multilobated (SPM)—Round cavities with small, fingerlike protuberances. As they were observed before on the antennae of the studied species, the name was given according to these other studies. The probable function is olfaction; however, olfactory structures are not specific to leg sensilla. Therefore, they might play another role.
9. Sensilla arch-shaped (SAr)—Thick, perpendicular to the surface of the legs, with obtusely rounded tips and striated surfaces.
10. Sensilla spoon-shaped (SSp)—Slender, cylindrical base adorned with stripes. The end expands into a spoon-like shape.

### 3.3. Sensilla Observed Among Representatives of the Studied Genera

#### 3.3.1. Sensilla on the Legs of *Anisops*

**Figure 3:**
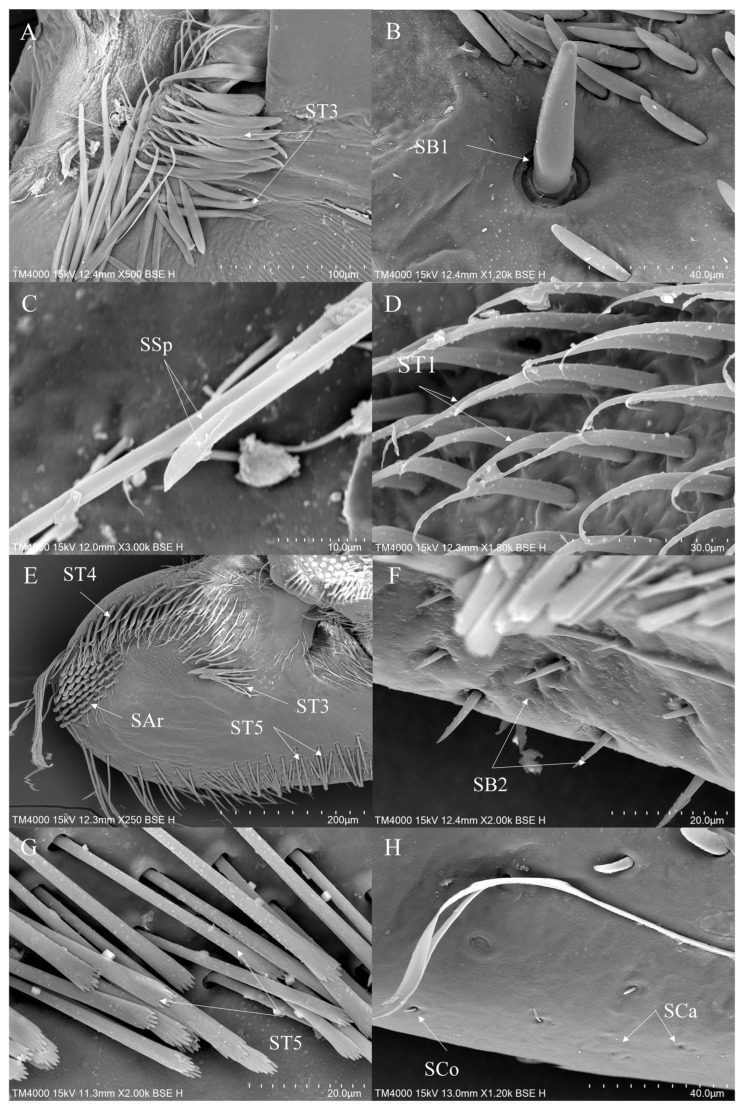
The foreleg sensilla of *Anisops*: *Anisops bouvieri* (**A**,**B**); *Anisops elstoni* (**C**–**F**); *Anisops stali* (**G**,**H**). SAr—sensilla arch-shaped, SB—sensilla basiconica, SCa—sensilla campaniformia, SCo—sensilla coeloconica, SSp—sensilla spoon-shaped and ST—sensilla trichodea.

**Figure 4:**
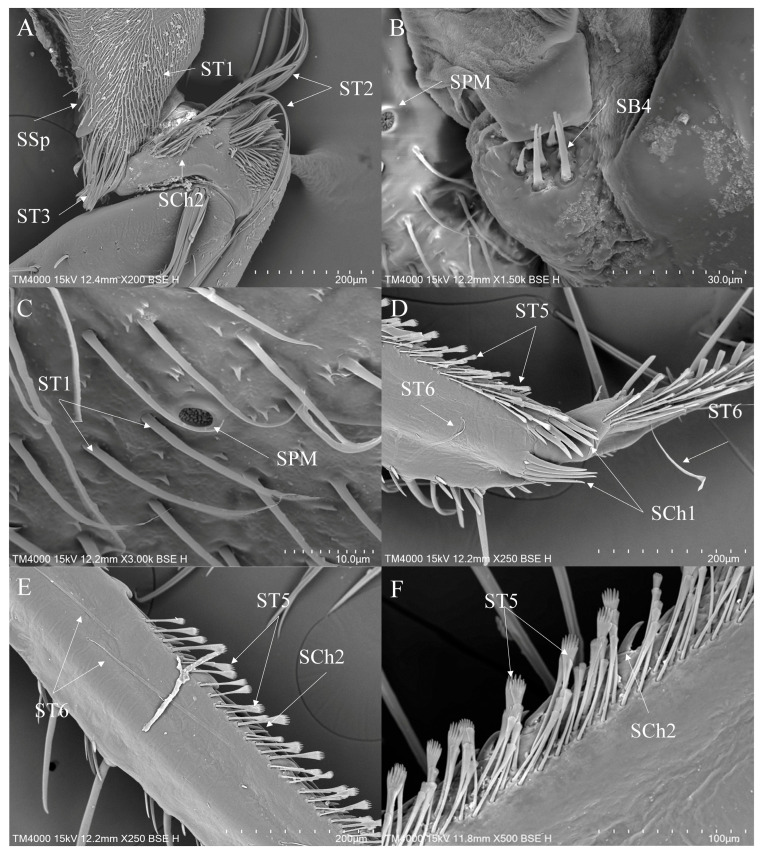
The midleg sensilla of *Anisops*: *Anisops bouvieri* (**A**); *Anisops elstoni* (**B**,**C**); *Anisops exiguus* (**D**); *Anisops kuroiwae* (**E**); *Anisops stali* (**F**). SB—sensilla basiconica, SCh—sensilla chaetica, SPM—sensilla placodea multilobated, SSp—sensilla spoon-shaped and ST—sensilla trichodea.

**Figure 5:**
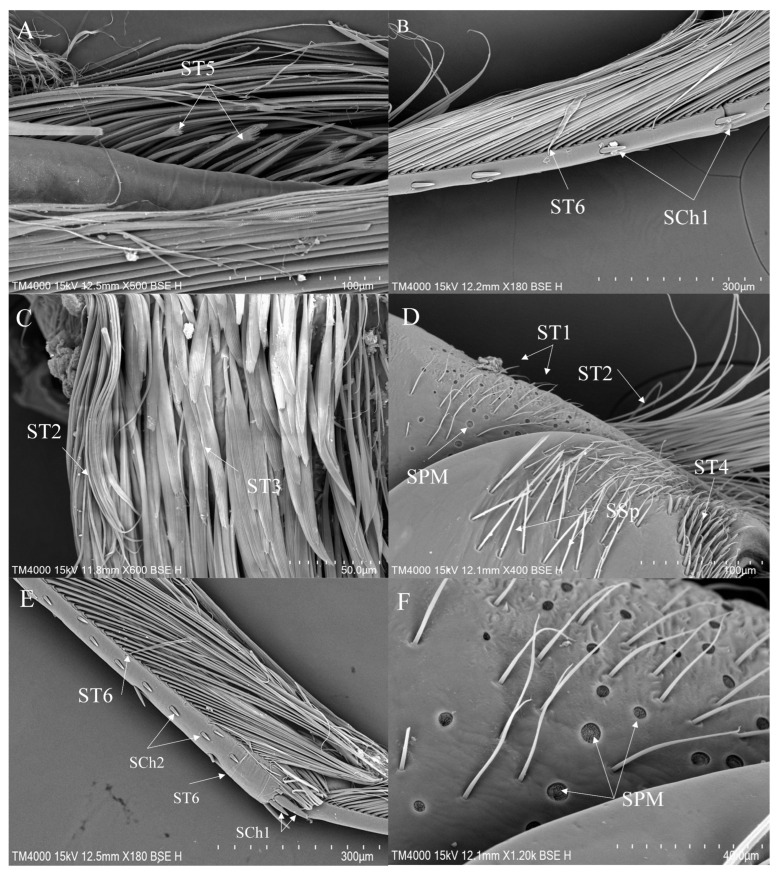
The hindleg sensilla of *Anisops*: *Anisops elstoni* (**A**–**C**); *Anisops stali* (**D**–**F**). SCh—sensilla chaetica, SPM—sensilla placodea multilobated, SSp—sensilla spoon-shaped and ST—sensilla trichodea.

ST1 is commonly present on the coxae and trochanters of all legs, covering most of the surface area of the coxae (Figure 3D, Figure 4A and Figure 5D). ST2 is usually distributed on the trochanters of the foreleg and the midleg (Figure 4A) and all segments of the hindleg except the tarsus (Figure 5C,D). ST3 is distributed on the trochanters of the foreleg (Figure 3A,E), the coxae of the midleg (Figure 4A) and the hindleg (Figure 5C). ST4 is typically distributed on the coxae and the trochanters of the foreleg (Figure 3E) and the hindleg (Figure 5D). ST5 is distributed on the trochanters and the femora of the foreleg (Figure 3E,G), the tibiae (Figure 4D,E), the tarsi (Figure 4F) of the midleg and the tarsi of the hindleg (Figure 5A). ST6 is distributed on the tibiae (Figure 4D,E and Figure 5E ) and the tarsi (Figure 5B) of all legs. SB1—unique to *Anisops*—occurs on the tibiae of the foreleg (Figure 3B). SB2 is distributed on all segments of the foreleg and the midleg except the coxae (Figure 3F). SB4 is typically distributed at the base of the trochanters on the midleg (Figure 4B). SCh1 is usually distributed on the femora and the distal ends of the tarsi (Figure 4D). SCh2 is widely distributed on the foreleg and the midleg (Figure 4A,E,F). SA belongs to the socketed peg-like sensilla, and its opening is the only part observable on the surface of the insect’s legs. Therefore, it is not easy to observe on the surface of the leg segments, but it has been observed on the foreleg (Table 2). SCa is a mechanoreceptive sensilla and is distributed on the tibiae of the foreleg (Figure 3H) and the hindleg.

SCo are observed on the tibiae of the foreleg (Figure 3H) and the hindleg. SPM are observed on the coxae of the midleg (Figure 4B,C) and the hindleg (Figure 5D,F). Additionally, a unique circular arched structure with longitudinal striations (SAr) was observed on the trochanters of the foreleg (Figure 3E). Based on its structural characteristics and position, this structure might be associated with spatial orientation within the complex aquatic environment or with the detection of water flow changes. SSp is present on the coxae of all legs (Figure 3C, Figure 4A and Figure 5D).

#### 3.3.2. Sensilla on the Legs of *Enithares*

**Figure 6:**
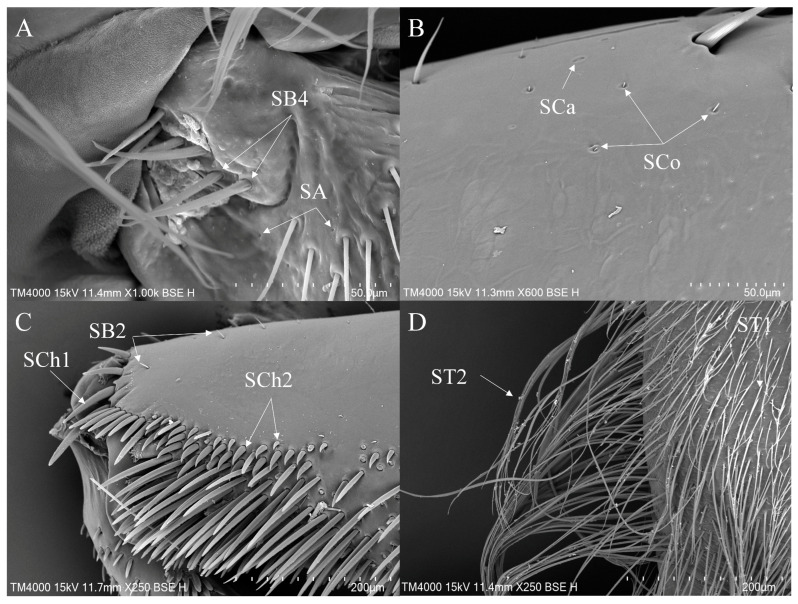
The foreleg sensilla of *Enithares*: *Enithares biimpressa* (**A**,**B**); *Enithares ciliata* (**C**,**D**). SA—sensilla ampullacea, SB—sensilla basiconica, SCa—sensilla campaniformia, SCh—sensilla chaetica, SCo—sensilla coeloconica and ST—sensilla trichodea.

**Figure 7:**
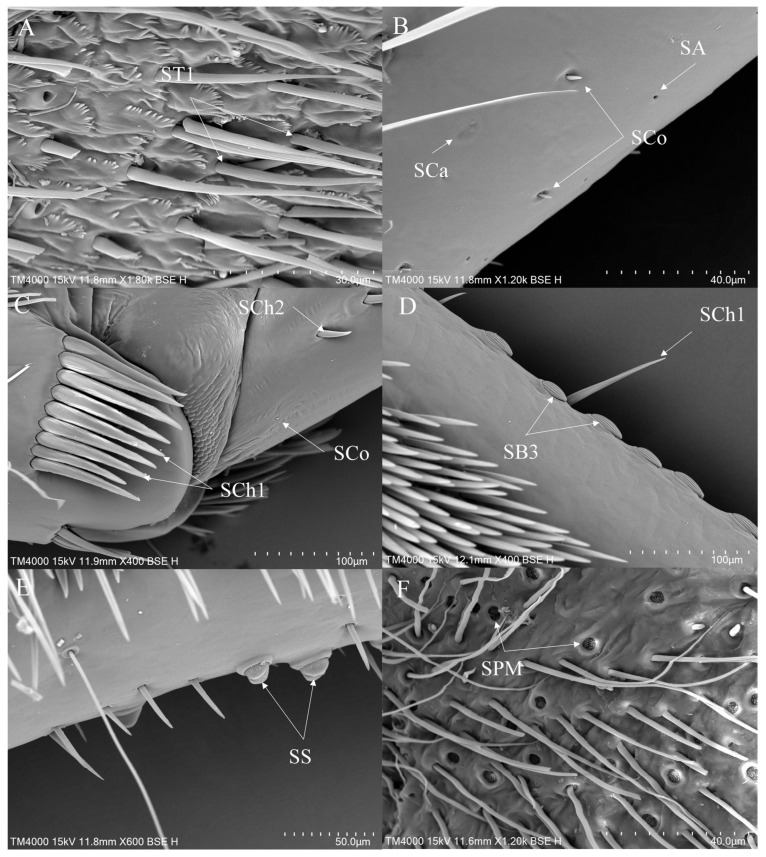
The midleg sensilla of *Enithares*: *Enithares biimpressa* (**A**–**D**); *Enithares sinica* (**E**,**F**); SA—sensilla ampullacea. SB—sensilla basiconica, SCa—sensilla campaniformia, SCh—sensilla chaetica, SCo—sensilla coeloconica, SPM—sensilla placodea multilobated, SS—sensilla styloconica and ST—sensilla trichodea.

**Figure 8:**
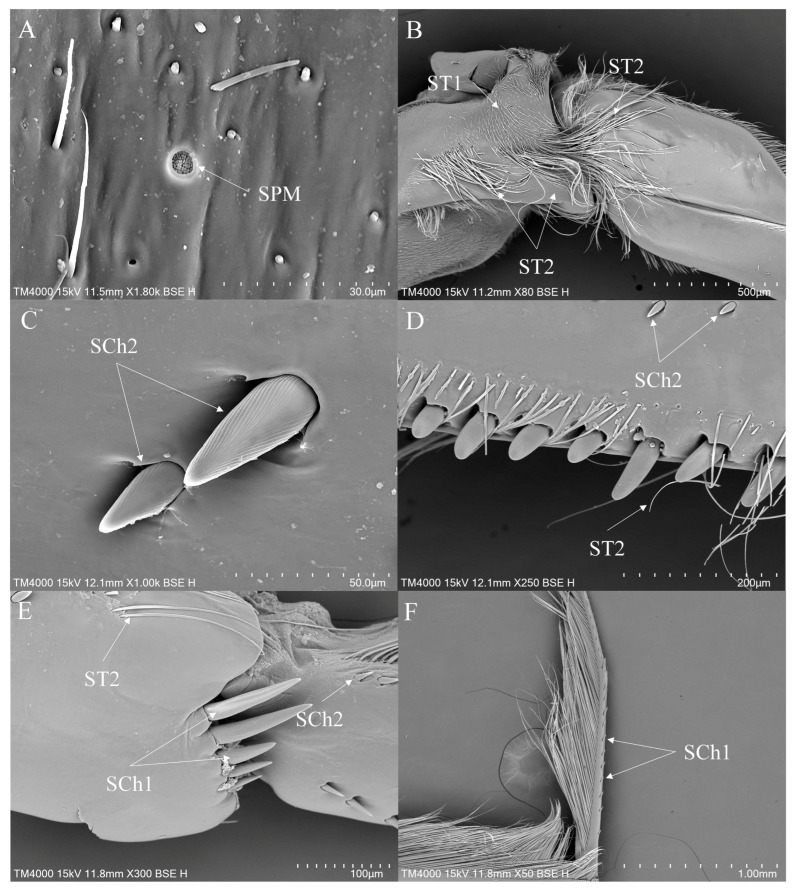
The hindleg sensilla of *Enithares*: *Enithares ciliata* (**A**); *Enithares sinica* (**B**,**C**); *Enithares tibialis* (**D**–**F**). SCh—sensilla chaetica, SPM—sensilla placodea multilobated and ST—sensilla trichodea.

Sensilla trichoidea are widely distributed on all segments of all legs in *Enithares*. ST1 is the most numerous subtype of sensilla trichoidea, distributed on the foreleg (Figure 6D), the midleg (Figure 7A) and the hindleg (Figure 8B). ST2 is mainly distributed on the coxae and the trochanters of all legs (Figure 6D and Figure 8B,E ). SB2 is distributed on the femora and the tibiae of the foreleg (Figure 6C). SB3 is distributed on the tibiae of the midleg (Figure 7D). SB4 is distributed on the trochanters of the foreleg (Figure 6A). SCh1 is prevalent on the femora, tibiae and tarsi of all legs (Figure 6C, Figure 7C and Figure 8E,F). SCh2 is commonly distributed on the trochanters, femora and tibiae of the foreleg (Figure 6C), the midleg (Figure 7C) and the hindleg (Figure 8C–E). Sensilla styloconica (SS) are observed on the ventral side of the femora of the midleg (Figure 7E). SA is small in size and not easy to observe. It is observed on the surfaces of the foreleg and the midleg (Figure 6A and Figure 7B).

SCa are typically distributed on the tibiae and the tarsi of all legs (Figure 6B and Figure 7B). SCo, is present on the femora, tibiae and tarsi of the foreleg (Figure 6B), the femora and tarsi of the midleg (Figure 7B,C). SPM is distributed on the coxae of the midleg (Figure 7F) and the hindleg (Figure 8A).

Additionally, the foreleg and the midleg have a greater variety of sensilla types, while the hindleg possesses fewer types, primarily sensilla trichoidea and sensilla chaetica (Table 2). Dense long setae are distributed on the ventral side of the tibiae and tarsi of the hindleg (Figure 8F).

#### 3.3.3. Sensilla on the Legs of *Notonecta*

**Figure 9:**
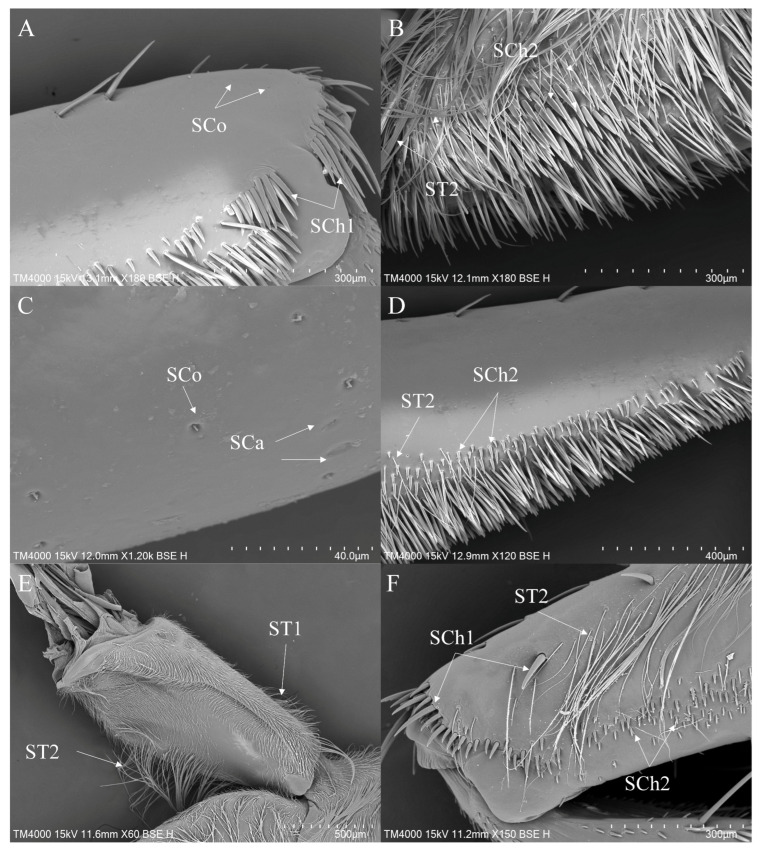
The foreleg sensilla of *Notonecta*: *Notonecta amplifica* (**A**,**B**); *Notonecta kiangsis* (**C**); *Notonecta montandoni* (**D**); *Notonecta triguttata* (**E**,**F**). SCa—sensilla campaniformia, SCh—sensilla chaetica, SCo—sensilla coeloconica and ST—sensilla trichodea.

**Figure 10:**
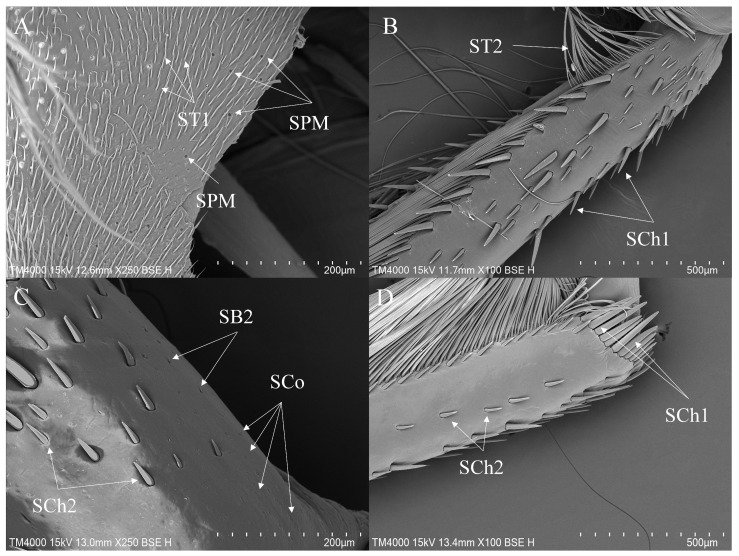
The hindleg sensilla of *Notonecta*: *Notonecta chinensis* (**A**,**B**); *Notonecta montandoni* (**C**); *Notonecta triguttata* (**D**). SB—sensilla basiconica, SCh—sensilla chaetica, SCo—sensilla coeloconica, SPM—sensilla placodea multilobated and ST—sensilla trichodea.

**Figure 11:**
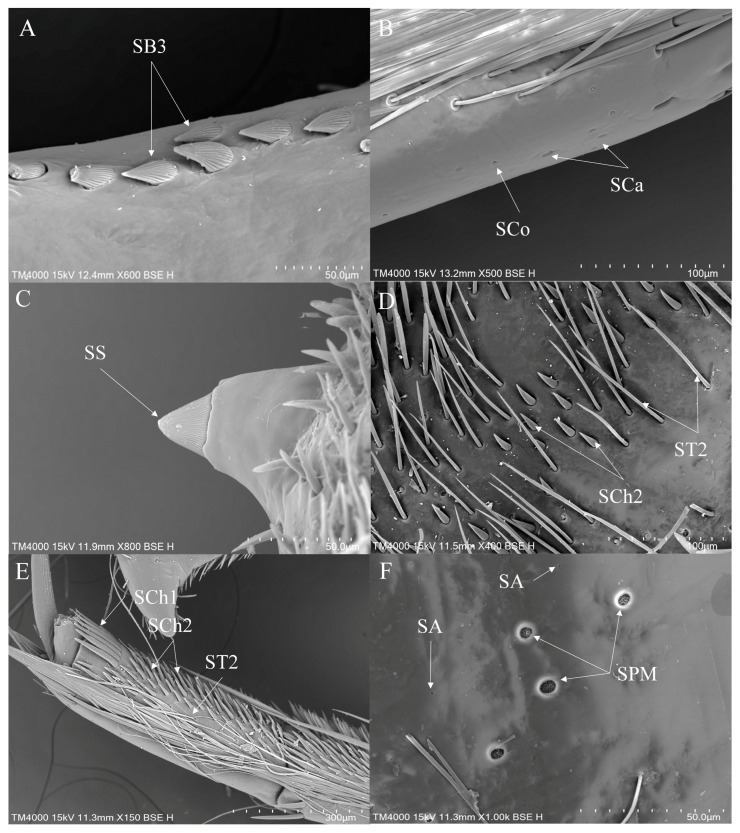
The midleg sensilla of *Notonecta*: *Notonecta amplifica* (**A**); *Notonecta chinensis* (**B**); *Notonecta kiangsi* (**C**,**D**); *Notonecta triguttata* (**E**,**F**). SA—sensilla ampullacea, SB—sensilla basiconica, SCa—sensilla campaniformia, SCh—sensilla chaetica, SCo—sensilla coeloconica, SPM—sensilla placodea multilobated, SS—sensilla styloconica and ST—sensilla trichodea.

In *Notonecta*, ST1 and ST2 are widely distributed sensilla types. ST1 is distributed on the coxae, trochanters and femora of all legs (Figure 9E and Figure 10A). ST2 is distributed on all segments of all legs (Figure 9E,F, Figure 10B and Figure 11E). SB are less commonly distributed in *Notonecta*. SB2 is distributed on the hindleg (Figure 10C). SB3 is typically distributed at the base of the tibiae of the midleg (Figure 11A). SCh1 is mainly distributed on the femora, tibiae and tarsi of all legs (Figure 9A,F, Figure 10B,D and Figure 11E). SCh2 is widely distributed on all legs. It occurs on all segments of the foreleg except the coxa (Figure 9B,D,F), on the femora and tibiae of the midleg (Figure 11D) and the femora of the hindleg (Figure 10D). SA are small in size, making them difficult to observe; they are observed on the foreleg and the midleg (Figure 11F). SCa are typically distributed on the tibiae and tarsi of all legs (Figure 9C and Figure 11B). SCo are distributed across the femora, tibiae and tarsi of the foreleg (Figure 9C) and the midleg (Figure 11B), as well as the femora of the hindleg (Figure 10C). SS are exclusively distributed on the ventral side of the femora of the midleg (Figure 11C). SPM are distributed on the coxae of the midleg and the hindleg (Figure 10A and Figure 11F).

## 4. Discussion

This paper presents the results of a morphological study on leg sensilla in seventeen species of Notonectoidae belonging to three genera, conducted with a scanning electron microscope. A total of ten main types of leg sensilla were identified. Previously, eight types of sensilla were identified in studies on the leg sensilla of Nepomorpha, including Gelastocoridae, Ochteridae and Corixidae [16]. In the present study, two new types of sensilla were discovered on the foreleg of *Anisops*.

Notonectidae primarily perceive prey through vision and surface vibrations [33]. Compared with the antennae, the legs possess a much greater diversity of sensilla types. This difference may be attributed to the division of labor in sensory functions among different organs: the antennae have a higher variety of chemoreceptors, whereas the legs exhibit greater diversity in mechanoreceptor types. From a morphological perspective, the antennae of Notonectidae are short and hidden under the head, leaving limited space for sensilla structures [26,33]. Due to their inability to provide a sufficient attachment area, the evolution of the number and types of antennae sensilla is structurally restricted. Compared with the antennae, the legs have a larger overall surface area, which provides sufficient “carrier space” for the diversification of sensilla. In previous studies, *Anisops* and *Notonecta* each had five types of sensilla on antennae, while *Enithares* had four types of sensilla [26]. In this study, a total of 10 types of sensilla were found on the legs of Notonectidae. Among them, nine types of sensilla were identified on the legs of *Anisops*, while eight types of sensilla were detected on the legs of both *Enithares* and *Notonecta*.

Five subtypes of sensilla basiconica were also observed on the antennae of Notonectidae [26]. The shape of each subtype differs from that of the legs. The sensilla basiconica (SB2–SB5) on the antennae usually have a porous surface and function as chemoreceptors, while SB1 has a smooth surface and serves as a proprioceptor. The types of thermo-hygroreceptors on the antennae are the same as those on the legs.

Differences exist among the studied genera. First, differences can be noted in leg shapes. The tibiae of *Anisops* are flattened, with the foreleg tarsi of males being unsegmented and those of females being two-segmented. In contrast, the tibiae of *Enithares* and *Notonecta* are cylindrical, and their tarsi are two-segmented.

Second, the leg sensilla observed in *Anisops* also differ from those of *Enithares* and *Notonecta*. Sensilla trichodea (ST) are additionally distributed on the antennae and labium across Nepomorpha species [27,35]. We found that ST are also common on legs. There are six subtypes of ST, among which ST1 and ST2 are the most common in *Enithares* and *Notonecta*. ST1-ST6 are distributed in *Anisops*. ST1 and ST2 are primarily responsible for mechanoreceptive functions, with the main difference between them lying in whether their surfaces are smooth. On the foreleg and the hindleg, ST5, with brush-like worn tips, and ST6, with rounded tips, were observed, which are presumed to be mechanoreceptors (Table 2). Given their position and length, they probably assist in swimming by increasing the surface area of the flattened leg segments [16]. Notably, ST6 occur exclusively on the tarsi of all legs in *Anisops*, where their elongated morphology may enhance hydrodynamic perception. Some studies also suggest ST may facilitate pheromone detection [15].

There are four subtypes of sensilla basiconica (SB) on legs (Table 2). SB1 is typically distributed on the tibiae of the foreleg of *Anisops*. SB2 occurs in all the studied genera, representing putative proprioceptors that detect segmental leg flexion. SB3 predominantly occurs along the tibia of the midleg of *Enithares* and *Notonecta*. Based on its morphology and distribution, this sensilla type may function as a mechanoreceptor, detecting hydrodynamic forces and pressure gradients to facilitate aquatic locomotion. SB4 is presumably specialized for chemoreception, transducing chemical cues.

There are two subtypes of sensilla chaetica (SCh). SCh1 and SCh2 are widely distributed on the legs of all the studied genera (Table 2). SCh1 and SCh2 likely serve mechanosensory functions, detecting external physical stimuli such as air currents and vibrations [23]. In Pleidae, these sensilla help detect environmental mechanical disturbances. When deflected by air currents or substrate vibrations, chaetica sensilla transduce physical stimuli into neural signals through hair shaft displacement, transmitting environmental information to the nervous system [36]. This transduction mechanism facilitates rapid behavioral responses. Furthermore, these sensilla likely play crucial roles in behaviors such as foraging, courtship and predator avoidance.

In *Enithares* and *Notonecta*, sensilla styloconica (SS) occur on the femora of the midleg. This sensilla type has also been documented in *Nerthra grandicollis* and *Nerthra mixta* [16]. The function of this sensilla may be a mechanoreceptor or a gustatory receptor. Additionally, styloconica sensilla are also found in specimens connected to the terrestrial environment [16].

Sensilla ampullacea (SA) is a type of thermo-hygrosensory structure. SA reside within sunken pits, exhibit lower abundance and consequently present observational challenges. In previous studies on the leg sensilla of Nepomorpha, SA were only observed on the leg surfaces of *Gelastocoris flavus*, with no specific restriction to a particular leg segment regarding their distribution position on the legs of this species.

Sensilla campaniformia (SCa) function as mechanoreceptors, enabling insects to detect mechanical stimuli and coordinate adaptive behavioral responses to environmental changes [23,24]. On legs, SCa are generally considered to respond directionally to cuticle bending during legged locomotion [37]. These structures are also documented in other Heteroptera groups [17,38]. SCa demonstrate ubiquitous distribution on insect bodies and have been hypothesized to serve proprioceptive functions in prior research [39,40].

Sensilla coeloconica (SCo) occur on antennae and legs throughout aquatic and terrestrial Hemiptera [12,23], where they function as thermo-hygroreceptors in all examined taxa. Their typically small dimensions and low abundance complicate observation. Insects can perceive temperature and humidity information in the environment, enabling them to better select a suitable living environment [24,25].

Sensilla placodea multilobated (SPM) were observed on the legs of the three genera. SPM were likewise present on Gelastocoridae and Ochteridae legs but absent from Corixidae legs [16]. Nowińska and Brożek proposed that its function is olfaction; however, olfactory structures are not exclusive to leg sensilla, so this type of sensilla may also serve additional functions. [16].

Another distinctive feature is the lack of porous sensilla on the legs of the studied specimens. This is to be expected, considering that olfactory sensilla are mostly present on the antennae and mouthparts of insects [41].

Our studies on Notonectidae are in line with the newest phylogenetic studies [42]. Phylogenetically, *Enithares* and *Notonecta* are sister groups, while *Anisops* belongs to the subfamily Anisopinae [42]. This explains why the sensilla of *Enithares* and *Notonecta* show little morphological difference, while the sensilla of *Anisops* differ significantly from them.

Although this study advances our understanding, several limitations warrant acknowledgment. Functional hypotheses regarding specific sensilla require experimental validation. Moreover, differences in the classification criteria of sensilla used in different studies have affected the comparison and integration of research results. Future research can utilize transmission electron microscopy to deeply explore the ultrastructure of sensilla, conduct functional experiments to clarify the functions of sensilla and unify the classification standards of sensilla, so as to gain a deeper understanding of the leg sensilla of Notonectidae.

## 5. Conclusions

This study systematically characterizes the morphological diversity and spatial distribution of leg sensilla in *Anisops*, *Enithares* and *Notonecta* using a scanning electron microscope (SEM).

Ten types of leg sensilla were observed in this study. Among them, sensilla basiconica (SB1, SB2) are presumably proprioceptors; sensilla trichodea (ST1–ST6), sensilla chaetica (SCh1, SCh2), sensilla campaniformia (SCa) and sensilla basiconica (SB3) are mechanoreceptors; sensilla styloconica (SS) are contact chemoreceptors and sensilla coeloconica (SCo) and sensilla ampullacea (SA) are thermo-hygroreceptors. In addition, two types of sensilla with unknown function (SPM and SB4) were also observed. Novel discoveries include the unique sensilla arch-shaped (SAr) located on the trochanters of the foreleg in *Anisops* and spoon-shaped sensilla (SSp) distributed on the coxae of all legs in *Anisops*.

The studied genera exhibit variations in leg sensilla. This study reveals differences in the leg mechanoreceptive sensilla between *Anisops*, *Enithares* and *Notonecta*. On the one hand, in *Anisops*, the unique types of mechanoreceptive sensilla include sensilla trichodea (ST5 and ST6). In contrast, the sensilla trichodea in *Enithares* and *Notonecta* usually only have two subtypes, named ST1 and ST2. Therefore, differences can be identified when comparing the species of the three genera. On the other hand, differences among *Anisops*, *Enithares* and *Notonecta* can also be observed when comparing sensilla other than mechanoreceptors. Styloconic sensilla (SS), which are thought to possibly have a gustatory function, are only observed in *Enithares* and *Notonecta*.

Compared with previous studies on the antennal sensilla of Notonectidae, the types of mechanoreceptors on the legs are more numerous. This is probably because Notonectidae mainly perceive prey through vision and mechanical vibrations, and the legs are the core organs for capturing prey.

## Figures and Tables

**Table 1:** List of examined species.

Family	Genus	Species	Number of Studied Specimens
Notonectidae	*Anisops*	*Anisops bouvieri* Kirkaldy, 1904	2
		*Anisops elstoni* Brooks, 1951	2
		*Anisops exiguus* Horváth, 1919	2
		*Anisops kuroiwae* Matsumura, 1915	3
		*Anisops ogasawarensis* Matsumura, 1915	2
		*Anisops stali* Kirkaldy, 1904	2
	*Enithares*	*Enithares biimpressa* (Uhler, 1860)	3
		*Enithares ciliata* (Fabricius, 1798)	2
		*Enithares sinica* (Stål, 1854)	2
		*Enithares tibialis* Liu et Zhang, 1991	2
	*Notonecta*	*Notonecta amplifica* Kiritshenko, 1930	2
		*Notonecta chinensis* Fallou, 1887	2
		*Notonecta kiangsis* Kirkaldy, 1897	2
		*Notonecta montandoni* Kirkaldy, 1897	2
		*Notonecta reuteri reuteri* Hungerford, 1928	2
		*Notonecta triguttata* Motschulsky, 1861	3
		*Notonecta violacea* Kirkaldy, 1897	2

**Table 2:** List of observed sensilla in the examined genera.

Sensillum Type	*Anisops*			*Enithares*			*Notonecta*		
	FL	ML	HL	FL	ML	HL	FL	ML	HL
ST1	+	+	+	+	+	+	+	+	+
ST2	+	+	+	+	+	+	+	+	+
ST3	+	+	+						
ST4	+		+						
ST5	+	+	+						
ST6	+	+	+						
SB1	+								
SB2	+	+	+	+					+
SB3					+			+	
SB4		+		+					
SCh1	+	+	+	+	+	+	+	+	+
SCh2	+	+		+	+	+	+	+	+
SS					+			+	
SA	+			+	+		+	+	
SCa	+		+	+	+	+	+	+	+
SCo	+		+	+	+		+	+	+
SPM		+	+		+	+		+	+
SAr	+								
SSp	+	+	+						

“+” means the presence of all types of sensilla found in the examined genera. FL—foreleg; ML—midleg; HL—hindleg.

## Data Availability

The original contributions presented in this study are included in the article. Further inquiries can be directed to the corresponding author.
